# Sleepiness, inflammation and oxidative stress markers in middle-aged males with obstructive sleep apnea without metabolic syndrome: a cross-sectional study

**DOI:** 10.1186/s12931-015-0166-x

**Published:** 2015-01-14

**Authors:** Daniela Kuguimoto Andaku, Vânia D’Almeida, Gláucia Carneiro, Sônia Hix, Sergio Tufik, Sônia Maria Togeiro

**Affiliations:** Department of Psychobiology, Universidade Federal de São Paulo (UNIFESP-EPM), São Paulo, SP Brazil; Department of Medicine, Universidade Federal de São Paulo (UNIFESP-EPM), São Paulo, SP Brazil; Department of Morphology and Physiology, Faculdade de Medicina do ABC–FUABC, Santo André, SP Brazil; Rua Napoleão de Barros, 925, CEP 04024-002 São Paulo, SP Brazil

**Keywords:** Obstructive sleep apnea, Excessive daytime sleepiness, Metabolic syndrome, Inflammation, C- reactive protein, Oxidative stress

## Abstract

**Background:**

The simultaneous occurrence of metabolic syndrome and excessive daytime sleepiness are very common in obstructive sleep apnea (OSA) patients. Both conditions, if present in OSA, have been reported to be associated with inflammation and disruption of oxidative stress balance that impair the cardiovascular system. To verify the impact of daytime sleepiness on inflammatory and oxidative stress markers, we evaluated OSA patients without significant metabolic disturbance.

**Methods:**

Thirty-five male subjects without diagnostic criteria for metabolic syndrome (Adult Treatment Panel III) were distributed into a control group (n = 10) (43 ± 10.56 years, apnea-hypopnea index - AHI 2.71 ± 1.48/hour), a non-sleepy OSA group (n = 11) (42.36 ± 9.48 years, AHI 29.48 ± 22.83/hour) and a sleepy OSA group (n = 14) (45.43 ± 10.06 years, AHI 38.20 ± 25.54/hour). Excessive daytime sleepiness was considered when Epworth sleepiness scale score was ≥ 10. Levels of high-sensitivity C-reactive protein, homocysteine and cysteine, and paraoxonase-1 activity and arylesterase activity of paraoxonase-1 were evaluated.

**Results:**

Patients with OSA and excessive daytime sleepiness presented increased high-sensitivity C-reactive protein levels even after controlling for confounders. No significant differences were found among the groups in paraoxonase-1 activity nor arylesterase activity of paraoxonase-1. AHI was independently associated and excessive daytime sleepiness tended to have an association with high-sensitivity C-reactive protein.

**Conclusions:**

In the absence of metabolic syndrome, increased inflammatory response was associated with AHI and daytime sleepiness, while OSA was not associated with abnormalities in oxidative stress markers.

## Background

A number of observational studies have suggested a strong association between obstructive sleep apnea (OSA) and cardiovascular disorders (CVD) [1-3]. Because obesity seems to be the major risk for both conditions, many studies have attempted to separate the effects of OSA on risk factors for CVD from those promoted by obesity [4-6]. Metabolic syndrome (MS) is a set of metabolic abnormalities that appears to stimulate the development of atherosclerotic diseases [7], and it may be an important confounder for cardiovascular consequences of OSA.

OSA associated with MS seems to lead to further cardiovascular dysfunction involved in the development of CVD. Greater inflammatory response [8,9], metabolic impairment [8], worse insulin resistance [10], higher sympathetic drive [11], increased arterial stiffness [9,12] and atherosclerosis [12] were detected in patients with both conditions. However, it is still unknown to what extent MS is responsible for these consequences.

The pathophysiological mechanisms that may contribute to the development of CVD in individuals with OSA arise from sleep fragmentation and intermittent hypoxia [13], which could lead to the symptom of excessive daytime sleepiness (EDS). This major symptom of OSA has been reported to be involved in the association of OSA and MS [14], even as EDS has also been reported to predict cardiometabolic complications in OSA patients [15-19]. Two case–control studies have found association between abnormalities on glucose metabolism and daytime sleepiness [18,19], and another study showed that only sleepy patients improved insulin resistance and cholesterol levels after CPAP treatment [19], evidencing the importance of this symptom on clinic evolution of OSA patients.

With regard to inflammation, higher levels of inflammatory markers were found in sleepy obese patients with OSA [20]. However, whether EDS is related to inflammation in patients with OSA without obesity or MS has not been well investigated. In this sense, it is reasonable to conceive that even in the absence of MS, EDS could be associated to greater inflammatory response in OSA patients.

We hypothesized that MS mediates OSA cardiovascular risk. Therefore, OSA patients without important metabolic dysfunction would not have major cardiovascular impairment, unless EDS is present. Thus, the aim of this study was to evaluate biochemical markers of inflammation and oxidative stress in OSA patients who do not fit into the diagnostic criteria for MS, as well as to investigate if EDS can influence these biochemical parameters.

## Methods

### Participants

The study included 11 male volunteers (control group) and 26 male patients with moderate to severe OSA selected from the Cardiovascular Metabolism Center and the Sleep Clinic at the Universidade Federal de Sao Paulo (UNIFESP). Moderate to severe OSA was defined as an apnea-hypopnea index (AHI) > 15/h. The control group was composed of subjects with AHI < 5/h. We distributed the OSA subjects into 2 groups according to excessive daytime sleepiness (non-EDS OSA and EDS OSA). All participants were asked to arrive at the laboratory in the early morning after 12 h of fasting to provide a blood a sample.

Subjects whose had body mass index (BMI) > 30 kg/m^2^, age >60 years, severe chronic pulmonary or cardiac diseases, diabetes mellitus (fasting glucose > 126 mg/dL) [21], MS according to the modified criteria of the National Cholesterol Education Program (NCEP) Adult Treatment Panel III (ATP III) [7] or previous OSA treatment were excluded. After data collection, 2 participants were excluded: 1 by diabetes mellitus diagnosis (from the control group), and 1 with MS (from the EDS OSA group).

The study was approved by the Ethics Committee for Research of the Universidade Federal de Sao Paulo (CEP1687/08) and was registered with ClinicalTrials.gov (NCT01635010). All participants gave written informed consent before being enrolled in the study.

### Polysomnography

All participants underwent an overnight polysomnography including electroencephalography (4 channels: C3-A2, C4-A1, O1-A2, O2-A1), electrooculogram (2 channels: LOC-A2, ROC-A1), electromyogram (2 channels: submental and anterior tibialis muscles), electrocardiogram (1 channel), and recording of snoring and body position. The airflow was monitored with a thermistor and a pressure cannula. Respiratory efforts were detected with ribcage and abdominal piezo sensors. Arterial oxygen saturation and pulse were monitored by pulse oximetry. Sleep scoring was conducted according to the criteria of Rechtschaffen and Kales [22]. Arousals were defined using criteria from the Sleep Disorders Atlas Task Force of the American Sleep Disorders Association [23]. Respiratory events, as well as OSA severity, were defined according to the American Academy Sleep Medicine Task Force [24]. The AHI was defined as the number of apneas and hypopneas per hour of sleep.

### Excessive daytime sleepiness

Daytime somnolence was evaluated subjectively using the Epworth sleepiness scale [25], with a score ≥10 considered excessive daytime sleepiness.

### Definition of metabolic syndrome

MS was diagnosed according to the NCEP ATP III modified criteria [7] which considers the occurrence of MS if three of the five following factors were present: 1) waist circumference ≥ 102 cm for men (≥90 cm for men of Asian origin); 2) triglycerides ≥ 150 mg/dL or patient on specific drug treatment; 3) HDL < 40 mg/dL for men or patient on specific drug treatment; 4) arterial blood pressure ≥ 130 or 85 mmHg respectively for systolic and diastolic blood pressure, or patient on antihypertensive drug treatment; 5) fasting glucose ≥ 100 mg/dL or patient on specific drug treatment.

### Blood sampling and biochemical marker determinations

Thirty milliliters of venous blood was obtained from each participant after a 12 h fasting period. The biochemical variables were high-sensitivity C-reactive protein (hs-CRP), homocysteine (Hcy) and cysteine (Cys) levels and paraoxonase-1 (PON-1) activity. As controls for the variables, we also analyzed fasting vitamins B_6_, B_12_ and folate, total cholesterol, LDL, HDL, very low-density lipoprotein cholesterol (VLDL), triglycerides, glucose, insulin, and 2 hour oral glucose tolerance test (2 h-GTT).

Serum hs-CRP determination was standardized according to the International Federation of Clinical Chemistry using an International Reference Preparation for Plasma Proteins (lot CRM 470) certified by the Bureau of Reference of the European Community. This method has been referred to as the Reference Preparation for Proteins in Human Serum lot 91/0619 from the College of American Pathologists [26]. Image CRPH reagent, which is based on high-sensitivity immunoassay methodology, was used.

Plasma Hcy and Cys levels were also measured as oxidative stress markers; their determination was based on the method described by Pfeiffer et al. [27] which uses high performance liquid chromatography with fluorimetric detection and isocratic elution. The activity of PON-1 was assessed by measurement of PON-1 paraoxonase activity (PON-1sal) and PON-1 arylesterase activity (PON-1are). These activities were measured by spectrophotometry using paraoxon and phenylacetate, respectively, as substrates [28]. Vitamin B_6_ in serum was quantified using high performance liquid chromatography with ultraviolet detection and isocratic elution [29]. Serum levels of vitamin B_12_ and folate were measured by chemiluminescent immunoassay with paramagnetic particles using the Access Immunoassay System (Beckman Coulter Inc., Brea, California, USA).

Measurement of total cholesterol was performed using an enzymatic colorimetric method. Calculations of LDL and VLDL were performed according to the Lipid Research Clinics Program [30]. Quantitative analysis of HDL was performed by the immunoinhibition method. The serum level of triglycerides was measured by the enzymatic colorimetric method using glycerol-3-phosphate-oxidase. The 2 h-GTT was administered in the morning after at least 10 h of fasting. Blood was collected after fasting and 120 min after oral ingestion of 75 g of glucose. Plasma glucose was determined using glucose oxidase calorimetric method. Insulin resistance was defined according to the Homeostasis Model Assessment-Insulin resistance (HOMA-IR) (fasting glucose [mg/dL] × fasting insulin [mU/mL]/405) > 2.7 [31]. Serum insulin was determined by radioimmunoassay.

### Statistical analysis

Categorical data were analyzed by Chi-squared test, with Fisher correction when appropriate, and expressed as absolute number (%). Due to the sample size, statistical analyses for all continuous values were performed using the Z score method for standardization of values, even though the data were normally distributed. Continuous variables were expressed as the mean (± standard deviation or standard error). Comparisons of continuous values among control, non-EDS OSA and EDS OSA groups were performed using one-way analysis of variance with Bonferroni *post hoc* test. An univariate general linear model for analysis of covariance was used to assess differences in hs-CRP, Hcy, Cys, PON-1sal, and PON-1are among the groups, with adjustment for waist circumference, HOMA-IR and triglycerides to avoid possible confounder effects. In this analysis, the inflammatory and oxidative stress markers were the dependent variables, the group category was the fixed factor, and the variables in the adjustments were covariates. Stepwise multiple linear regression analysis was performed to evaluate the influence of AHI, EDS and age on hs-CRP levels. All analyses were conducted using SPSS software for Windows, version 13.0 (Chicago, USA). Values of p < 0.05 were considered statistically significant.

## Results

The clinical characteristics of the control, non-EDS OSA and EDS OSA groups are presented in Table 1. The groups were similar with respect to age, fasting glucose, 2 h-GTT and total cholesterol, HDL and LDL levels (Table 1). The Epworth sleepiness scale score was significantly higher in the EDS OSA group than in the control group (p < 0.001) and the non-EDS OSA group (p < 0.001) (Table 1). AHI was elevated in both non-EDS OSA and EDS OSA subjects (p < 0.01; p < 0.001, respectively), MinSatO_2_ was lower (p < 0.05; p < 0.01, respectively), and arousal index was higher in EDS-OSA subjects (p < 0.05) than in control subjects, all of these parameters did not differ significantly between the OSA groups (Table 1). The EDS OSA group presented elevated BMI (p < 0.01), waist circumference (p < 0.05), HOMA-IR (p < 0.05), VLDL (p < 0.05) and triglyceride levels (p < 0.05) compared to control subjects (Table 1). There was no difference in the number of MS components presented by the groups (Table 1).

**Table 1:** **Characteristics of control, non-EDS OSA and EDS OSA groups**

	**CONTROL (n = 10)**	**non-EDS OSA (n = 11)**	**EDS OSA (n = 14)**
**Age (years)**	43.00 ± 10.56	42.36 ± 9.48	45.43 ± 10.06
**BMI (kg/m** ^**2**^ **)**	24.14 ± 2.67	26.65 ± 2.38	27.39 ± 2.05*
**Waist Circumference (cm)**	89.10 ± 8.78	95.27 ± 8.04	97.93 ± 4.51*
**AHI (events/h)**	2.71 ± 1.48	29.48 ± 22.83*	38.20 ± 25.54*
**Arousal index (events/h)**	10.69 ± 5.79	25.91 ± 18.87	32.50 ± 20.53*
**MinSatO**_**2**_ **(%)**	90.70 ± 2.83	81.91 ± 8.27*	81.50 ± 7.51*
**Epworth sleepiness scale**	6.90 ± 2.02	6.55 ± 2.11	15.93 ± 4.44*^#^
**Fasting Glucose (mg/dL)**	86.80 ± 4.54	93.09 ± 9.58	92.71 ± 11.18
**2 h-GTT (mg/dL)**	97.00 ± 25.23	121.73 ± 39.01	109.21 ± 25.89
**HOMA-IR**	0.76 ± 0.31	1.82 ± 1.52	2.02 ± 1.16*
**Total Cholesterol (mg/dL)**	178.80 ± 40.42	190.73 ± 38.75	191.50 ± 25.63
**HDL (mg/dL)**	53.60 ± 17.20	52.00 ± 9.58	45.57 ± 7.94
**LDL (mg/dL)**	109.80 ± 28.89	117.45 ± 33.65	120.43 ± 26.06
**VLDL (mg/dL)**	15.30 ± 8.15	20.36 ± 8.16	25.50 ± 8.16*
**Triglycerides (mg/dL)**	77.10 ± 41.13	101.82 ± 41.53	128.07 ± 40.64*
**0 component of SM [n, (%)]**	5 (50.00)	3 (27.27)	4 (28.57)
**1 component of SM [n, (%)]**	3 (30.00)	5 (45.45)	5 (35.71)
**2 components of SM [n, (%)]**	2 (20.00)	3 (27.27)	5 (35.71)

BMI = body mass index; AHI = Apnea-Hypopnea Index; MinSatO_2_ = Minimum Oxygen Saturation; 2 h-GTT = 2 h oral glucose tolerance test; HOMA-IR = homeostasis model assessment index; HDL = High-density lipoprotein; LDL = Low-density lipoprotein; VLDL = Very-low-density lipoprotein. Data are presented as the mean ± SD or n (%). *ANOVA or Chi-squared*. *Different from control (p < 0.05). ^#^Different from non-EDS OSA group (p < 0.05).

To avoid possible confounding effects on the studied markers, analysis of covariance was performed adjusting for waist circumference, HOMA-IR and triglycerides. The hs-CRP levels of EDS OSA subjects were higher than those of the other groups (p < 0.05 for both) even after adjustments for confounders (Table 2). PON-1sal and PON-1are activities were similar in the three groups (Table 2). Plasma Hcy levels were lower in both the non-EDS OSA (p < 0.05) and the EDS OSA (p < 0.005) groups than in the control group, and plasma Cys levels were lower in EDS OSA subjects (p < 0.05) than in controls (Table 2). There were no significant differences in vitamin B_6_, B_12_ or folate levels among the groups (Table 2).

**Table 2:** **Inflammatory and oxidative stress markers of control, non-EDS OSA and EDS OSA groups**

	**CONTROL (n = 10)**	**non-EDS OSA (n = 11)**	**EDS OSA (n = 14)**
**Hs-CRP (mg/dL)**	0.11 ± 0.08	0.21 ± 0.06	0.41 ± 0.06*^#^
**PON-1 sal (U/mL)**	200.47 ± 44.09	202.08 ± 36.15	243.03 ± 35.15
**PON-1 are (U/mL)**	77.20 ± 9.95	90.96 ± 8.16	81.47 ± 7.93
**Hcy (mol/L)**	15.09 ± 1.31	10.75 ± 1.07*	9.28 ± 1.04*
**Cys (mol/L)**	596.59 ± 29.84	526.21 ± 24.47	485.49 ± 23.80*
**B**_**6**_ **(nmol/L)**	22.91 ± 5.15	25.56 ± 6.12	27.30 ± 8.20
**B**_**12**_ **(pg/mL)**	368.50 ± 184.12	304.45 ± 87.08	324.64 ± 121.23
**Folate (ng/mL)**	9.59 ± 4.04	10.95 ± 3.73	10.79 ± 3.51

Hs-CRP = high-sensitivity C-reactive protein; PON-1 sal = PON-1 paraoxonase activity; PON-1 are = PON-1 arylesterase activity; Hcy = Homocysteine; Cys = Cysteine. Data are presented as the mean ± SD or SE. *Analysis of covariance adjusted for waist circumference, HOMA-IR and triglycerides*. *Different from control (p < 0.05). ^#^Different from non-EDS OSA group (p < 0.05).

Stepwise multiple linear regression analysis showed that AHI was independently associated with hs-CRP (β = 0.314; p < 0.05); and excessive daytime sleepiness tended to have an association with hs-CRP (β = 0.245; p = 0.052).

## Discussion

In the present study we were able to demonstrate that middle-aged males with moderate to severe OSA, without significant metabolic dysfunction, had no abnormalities on studied oxidative stress markers. However, increased inflammatory response was found in OSA patients who exhibited daytime sleepiness.

The relationship between OSA, obesity and MS with inflammation has been explored in the literature. Studies have demonstrated an association between CRP and obesity [5,32,33], between CRP and MS [9], or between other inflammatory markers and visceral obesity [34] in OSA patients.

However, the impact of isolated OSA on inflammation is controversial. Punjabi & Beamer [35] and Kokturk et al. [36] showed significant associations among CRP levels and OSA in patients without CVD. In a recent study including non-obese males, hs-CRP, as well as interleukin-6 levels, were higher in OSA subjects compared to controls [6]. We demonstrated on multiple linear regression model, that AHI and EDS were independently associated with hs-CRP. Even after controlling for confounding factors, our patients with EDS also presented increased hs-CRP levels.

To the best of our knowledge, the finding that EDS was independently associated with inflammation in isolated OSA had not yet been well demonstrated. Additionally, we have to emphasize that there is growing evidence in literature that somnolence in OSA has a negative influence on cardiometabolic parameters [15-19], and this symptom predict positive therapeutic response to CPAP for many outcomes variables [19]. So, even that other conditions such as metabolic dysfunctions, obesity or cardiovascular disease that could increase inflammation are not present in OSA, the occurrence of EDS could be a marker of inflammation, also reported in our study. In this sense, our results are in agreement with Vgontzas et al. data [20], which informed an association of EDS with inflammatory markers. In opposite, Drager et al. [8], Bonsignore et al. [10] and Young et al. [37] did not find association of EDS with inflammatory markers [8], prevalence of MS components, insulin resistance [10], neither with death rate [37]. We have to take into account that these studies did not exclude metabolic and cardiovascular diseases, conditions that could have a major impact on the research outcomes.

We also should stress out that such differences can also be partially justified by the subjective measurement of EDS. Since the multiple sleep latency test - the objective measurement of sleepiness - has not been routinely included in the workup of these patients. In addition, sleep deprivation might be a confounder related to somnolence, and if sleep deprivation is also independently associated with inflammation needs to be studied.

Atherogenesis is related to inflammation and to oxidative stress biomarkers. The PON-1 activity has a protective role [38], while higher Hcy levels are considered an independent risk factor for atherosclerosis [39]. Few studies have addressed PON-1 activity in OSA patients. Lavie et al. [38] measured PON-1are activity in patients with OSA plus CVD, patients with OSA without CVD, and controls. Although stepwise regression analysis demonstrated an independent significant negative association between AHI and PON-1are activity, PON-1are activity was lower in the OSA plus CVD group than in controls, and tended to be lower in the OSA plus CVD group than in the OSA without CVD group. There were no differences between OSA without CVD and control groups, corroborating our findings, in which the exclusion of CVD and MS resulted in no significant differences in PON-1 activity among the groups, indicating that OSA itself is not associated with alterations on PON-1 activity. We should take in mind that we can not rule out that other antioxidants enzymes such as superoxide dismutase and catalase have participated in prevent the oxidative stress, however these were not analyzed in our study. Considering our results we could suggest that OSA patients were less suitable to disruption of oxidative balance than those with CVD pointing to the relevance of oxidative stress in the worsening of apnea syndrome.

Our findings also showed that Hcy and Cys levels were lower in the OSA group than in the control group. Although the mean values of plasma Hcy were within the normal range in control and OSA groups (<15 μMol/L [40]); four subjects (40%) in the control group (two of them with systemic hypertension), and two subjects (8%) in the OSA group presented hyperhomocysteinemia. In fact, even if the literature is controversy regarding the significance of Hcy levels in OSA, it seems that high levels of Hcy are observed when OSA is associated with CVD [41,42]. Excluding the presence of CVD, as in our study, Ryan et al. [33] and Lavie et al. [42] did not find elevated Hcy levels related to OSA.

Although statistical treatment was performed to accomplish our purpose, one limitation of our study was the lack of homogeneity of the groups with respect to BMI, centralization of fat, insulin resistance and lipid levels. It should be considered that in subjects with OSA and sleepiness, even without diagnostic criteria for MS, this observed tendency to develop metabolic dysfunctions and central obesity pattern in combination with increased inflammatory response may be a manifestation of a future MS. Kono et al. [43] also showed that even in non obese patients, OSA may predispose to the development of MS. Another recent study found similar results and suggested that OSA is a manifestation of an underlying MS [6].

Further aspects of this study should be taken into account when interpreting our results: i) the exclusion of women from the sample; ii) the small sample size (this was due to the difficulty in selecting subjects with OSA and without MS or obesity); iii) the design of the study (cross-sectional study) does not allow to demonstrate a causal relationship between OSA and CVD. On the other hand, most previous studies have investigated the effects of the association of OSA and MS on the cardiovascular system. In the current study, we were able to separate these two conditions, contributing to better understand the cardiovascular risk of OSA.

## Conclusions

In summary, we have shown that moderate to severe OSA, in the absence of significant metabolic dysfunction, did not affect the levels of the studied oxidative stress markers, while increased inflammatory response was associated with AHI and daytime sleepiness. These findings may contribute to understand better the pathophysiology as well as the importance of recognizing the somnolent phenotype of OSA.

## Abbreviations

2 h-GTT: 2 hour glucose tolerance test
AHI: Apnea-hypopnea index
ATP III: Adult treatment panel III
BMI: Body mass index
CVD: Cardiovascular disorders
Cys: Cysteine
EDS: Excessive daytime sleepiness
Hcy: Homocysteine
HOMA: Homeostasis model assessment
hs-CRP: High-sensitivity C-reactive protein
IR: Insulin resistance
MinSatO_2_: Minimum oxygen saturation
MS: Metabolic syndrome
NCEP: National Cholesterol Education Program
OSA: Obstructive sleep apnea
PON-1are: Paraoxonase-1 arylesterase activity
PON-1sal: Paraoxonase-1 activity
